# Epidemiological and clinical aspects of autoimmune thyroid diseases in children with Down’s syndrome

**DOI:** 10.1186/s13052-018-0478-9

**Published:** 2018-03-21

**Authors:** Tommaso Aversa, Giuseppe Crisafulli, Giuseppina Zirilli, Filippo De Luca, Romina Gallizzi, Mariella Valenzise

**Affiliations:** Department of Human Pathology in Adulthood and Childhood, University of Messina, Via Consolare Valeria, 98124 Messina, Italy

**Keywords:** Extra-thyroidal autoimmunity, Graves’ disease, Hashimoto’s thyroiditis, Metamorphic autoimmunity, Thyroid status

## Abstract

Aim of this commentary is to report the main peculiarities that have been found to characterize the phenotypic expression of autoimmune thyroid diseases (AITDs) in children with Down’s syndrome (DS). According to recent reports, DS children are, per se, more exposed to the risk of both Hashimoto’s thyroiditis (HT) and Graves’ disease (GD), irrespective of other concomitant risk factors, such as female gender and family antecedents for AITDs. In the context of extra-thyroidal autoimmune disorders, the ones that preferentially aggregate with AITDs in DS children are alopecia areata and vitiligo. Another peculiar aspect, in DS children, is that HT presents with a more severe biochemical picture, which furtherly deteriorates over time. By contrast, GD does not demonstrate a more severe clinical and biochemical picture with respect to that generally observed in patients without DS. Finally, DS children might be at higher risk of progressing from HT toward GD over time.

## Background

With a prevalence of 1:800 live births [1], Down’s syndrome (DS) is the commonest chromosomopathy in humans and the most frequent cause of severe learning disabilities [2].

One of the most typical clinical features of DS children is their susceptibility toward several autoimmune diseases, such as Hashimoto’s thyroiditis (HT), Graves’ disease (GD), type 1 diabetes, celiac disease, alopecia, vitiligo and idiopathic arthritis [2, 3]. Furthermore, the association with DS seems to be able to condition, per se, an over-expression of autoimmune phenomena [3], as suggested by both the non-exceptional co-occurrence of many autoimmune disorders [4–6] and the spontaneous progression from HT to GD, that has been reported to occur more frequently in children with this syndrome [7, 8] than in the pediatric general population [9]. These findings as a whole provide insights into a very aggressive phenotypic expression of autoimmunity in DS children [3, 10].

Another peculiar aspect of autoimmunity in DS children is that the association with this syndrome could modify the clustering of extra-thyroidal autoimmune disorders [3].

Aim of this commentary is to report the current views about the phenotypic peculiarities of DS-related autoimmune thyroid disorders (AITDs), in terms of epidemiology and pathophysiology, presentation, clinical and biochemical course and long-term metamorphic evolution.

### Epidemiology and pathophysiology

In DS children, HT is, by far, the most common autoimmune disease ad its prevalence has been reported to be much more elevated than that generally reported in age-matched patients without this chromosomopathy: respectively 13–34% [11, 12] vs 1.3% [13].

In DS children, also the prevalence of GD is known to be higher than in the pediatric general population: respectively 6.5‰ [14] vs 1.07‰ [15].

In patients with this syndrome both HT and GD do not show any gender predominance [8, 16], whilst in individuals without DS they are frequently associated with female sex. Furthermore, the frequency of AITD family antecedents in the history of DS children with either HT [8] or GD [16] is very low, which is atypical and surprising. Both these findings suggest that children with DS are, per se, more prone to the risk of developing AITDs, irrespective of other concomitants risk factors [8, 16, 17].

Another peculiar aspect of AITDs in DS children is that both HT and GD present at a younger age than in the pediatric general population [8, 11, 14, 16, 17]. This finding might be explained by the fact that several pediatricians are aware that DS children are more exposed to the risk of developing concomitant thyroid disorders and, therefore, the observation of a thyroid enlargement or dysfunction in a DS child is probably regarded with greater attention [8].

According to the results of a recent study, the prevalence rate of extra-thyroidal autoimmune disorders in DS children seems to be distinctly higher than in a control population of children without DS [3]. These findings confirm the non-casual association between DS and various extra-thyroidal autoimmune disorders, which had been previously postulated also by other authors [18–20]. In the study by Aversa et al. [3], the epidemiological distribution of extra-thyroidal autoimmune diseases was significantly different in two patient cohorts with or without DS. In particular, the prevalence rates of alopecia areata ad vitiligo were significantly more elevated in DS patients, whilst the prevalence of type 1 diabetes did not differ in the two groups and that of celiac disease became significantly more frequent only in the older patients of DS series [3]. The results of such study suggest that AITDs in DS children aged between 1 and 18 years demonstrate a preferential clustering with alopecia areata and vitiligo, irrespective of age [3].

In DS patients it is the organ-specific autoimmunity to be especially enhanced, whereas generalized autoimmunity is not remarkably represented [21]. Therefore, the relationships between DS and autoimmunity might be interpreted on the basis of a functional impairment of T cells, due to insufficient intrathymic expression of the AIRE gene [22], that is located on chromosome 21 and plays an important role in maintaining a balance between autoreactivity and immunoregulations in human autoimmunity [23]. According to this hypothesis, the thymus of children with DS might contain lower levels of the AIRE gene, which can account for both the altered expression of autoimmune regulation and the peculiar autoimmune phenotype of DS patients [3, 24].

### AITD presentation

Thyroid function patterns at HT presentation in children and adolescents without DS are known to range from overt hypothyroidism to overt hyperthyroidism [25]. The biochemical picture that is detected most often, at HT diagnosis, is euthyroidism, followed by either overt or subclinical hypothyroidism (SH) or, occasionally, overt and subclinical hyperthyroidism [26].

**Fig. 1:**
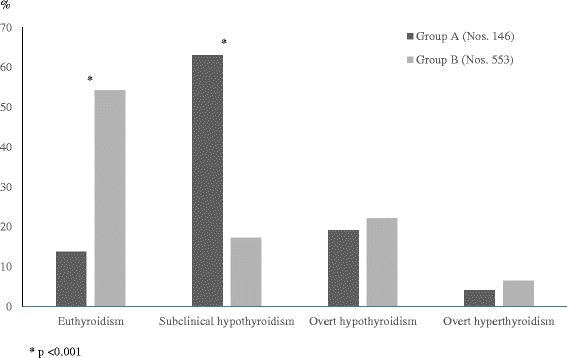
Prevalence rates (%) of the biochemical pictures of thyroid function found, at diagnosis of Hashimoto’s thyroiditis (HT), in two cohorts of HT children with (Group A) or without Down’s syndrome (Group B) (according to the results of Reference [8] study)

By contrast, in DS children the most common biochemical pattern, at HT presentation, is by far SH, followed by overt hypothyroidism, euthyroidism and hyperthyroidism, in decreasing order [17]. The prevalence of euthyroidism, at HT diagnosis, is significantly lower in DS children than in those without DS (Fig. 1).

The more severe biochemical presentation of HT in DS children might be interpreted on the light of a congenital alteration in thyroid gland regulation, that is peculiar of the individuals with this syndrome, irrespective of autoimmunity [27].

By contrast, the clinical presentation of GD in DS children does not seem to differ from that observed in children without DS [14, 16]. In fact, at GD presentation, the prevalence rates of both exophthalmos and other hyperthyroid manifestations were found to be very similar in DS children and controls [14, 16]. Furthermore, mean FT4 and median TSH receptor autoantibody (TRAB) serum levels did not differ in the two groups and the initial methimazole dosage that was needed in DS patients at the start of therapy, was not different from the one employed in GD children without DS [16].

All these findings as a whole are consistent with the view that GD in DS children does not present with a more severe clinical and biochemical picture.

### AITD course

**Fig. 2:**
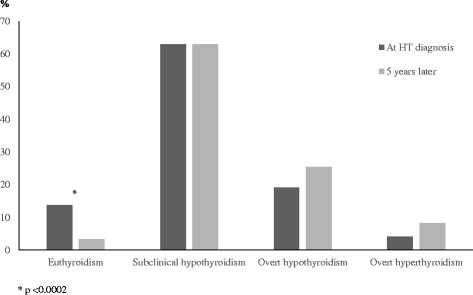
Prevalence rates (%) of the biochemical pictures of thyroid function found, both at diagnosis of Hashimoto’s thyroiditis (HT) and 5 years later, in a cohort of 146 children with Down’s syndrome (according to the results of Reference [8] study)

The natural evolution of thyroid status in 146 DS children with HT has been recently investigated throughout a 5-year follow-up [8]. According to the results of that longitudinal study, natural history of thyroid function in DS children with HT seems to be characterized by a progressive deterioration over time, as suggested by the significant decrease in the prevalence rate of euthyroidism from HT diagnosis onward (Fig. 2). As an obvious consequence of such a decrease, almost the totality of DS patients exhibited, 5 years after HT presentation, a hormonal pattern compatible with thyroid dysfunctions: either SH or overt hypothyroidism or overt hyperthyroidism (Fig. 2). A deterioration of thyroid function over time was also observed in another longitudinal study in DS children with HT-related SH [28]. Therefore, on the light of the results of these longitudinal studies [8, 28], it may be inferred that a prolonged follow-up of thyroid status is necessary in all the DS patients with HT, even in those presenting with euthyroidism and not only in those presenting with SH.

In the study by De Luca et al. [16] on the peculiarities of GD in young patients with DS, the most striking differences between DS children and those without DS concerned clinical response to pharmacological treatment. In fact, DS patients exhibited both lower relapse rates during the first methimazole cycle and higher remission rates after definitive methimazole withdrawal [16]. Furthermore, the methimazole dosages which were needed to maintain euthyroidism under treatment were lower in DS group [16]. It may be argued, on the light of these findings, that clinical course of GD is less severe in children with DS than in those without DS [16]. Such inference is reinforced by the observation that alternative non-pharmacological therapies were never requested in any DS children [16].

To sum up, whilst the course of HT in the children with DS has been repeatedly reported to be more severe that in age-matched individuals [8, 28], the course of DS-related GD seems to be relatively mild [16]. Therefore, it may be inferred that the association with DS might have a dichotomic impact on the phenotypic expression of these autoimmune disorders.

### Long-term metamorphic evolution

The metamorphosis of clinical phenotype from HT to GD or vice versa has been, during the last 10 years, the theme of many reports, which concluded that there exists a continuum between these disorders within the spectrum of AITDs [29, 30].

However, whereas the progression from HT to GD in the pediatric general population involves only 3.7% of children [9], this metamorphosis seems to be by far more frequent in the children with DS: 25% of cases [31]. On the light of these findings, it was suggested that DS children with concomitant HT might be at higher risk of progressing toward GD [7, 8, 17, 31], although the pathophysiological bases of such a predisposition were not elucidated [32].

## Conclusions

1) DS children are, per se, more exposed to the risk of AITDs, irrespective of other concomitant risk factors; 2) the extra-thyroidal autoimmune disorders which cluster most frequently with AITDs in DS children are alopecia areata and vitiligo; 3) in DS children, HT presents with a more severe biochemical picture, that furtherly deteriorates over time; 4) by contrast, GD presentation and course are not more severe in DS children than in those without DS.

## Abbreviations

AITDs: Autoimmune thyroid disorders
DS: Down syndrome
GD: Graves’ disease
HT: Hashimoto’s thyroiditis
SH: Subclinical hypothyroidism
TRABs: TSH receptor autoantibodies
